# The Association of Anti-dsDNA Antibodies with Patient-reported Outcomes of Patients with Systemic Lupus Erythematosus in a Two-consecutive Year Prospective Study

**DOI:** 10.24546/0100492949

**Published:** 2025-02-03

**Authors:** YUTO NAKAKUBO, HIDEAKI TSUJI, YUDAI TAKASE, TSUNEYASU YOSHIDA, TOMOHIRO KOZUKI, TAKESHI IWASAKI, MIREI SHIRAKASHI, HIDEO ONIZAWA, RYOSUKE HIWA, KOJI KITAGORI, SHUJI AKIZUKI, RAN NAKASHIMA, AKIRA ONISHI, HAJIME YOSHIFUJI, MASAO TANAKA, AKIO MORINOBU

**Affiliations:** 1 Department of Rheumatology and Clinical Immunology, Graduate School of Medicine, Kyoto University, Kyoto, Japan; 2 Department of Advanced Medicine for Rheumatic Diseases, Graduate School of Medicine, Kyoto University, Kyoto, Japan

**Keywords:** Anti-DNA antibody, Autoantibody, Patient reported outcome, Systemic lupus erythematosus

## Abstract

**OBJECTIVES:**

To analyse the association of anti-dsDNA Ab with patient-reported outcomes (PROs) in patients with SLE under maintenance treatment, as it has not been clearly understood whether PROs are able to be reflected by anti-dsDNA, which are associated with SLE activities (SLE Disease Activity Index 2000, SLEDAI-2K).

**METHODS:**

The SLE symptom checklist (SSC), LupusPRO, Medical Outcomes Study Short Form-36 (SF-36), and patient and physician visual analogue scale (Pt/Ph-VAS) at a time point were evaluated for correlation with anti-dsDNA using the Kyoto Lupus Cohort Registry (n = 310) from 2019 to 2020. Further, associations between changes in anti-dsDNA with those in Pt/Ph-VAS and SSC at two time points of short-term (3 months) or long-term (2 years) time points.

**RESULTS:**

Cross-sectionally, anti-dsDNA slightly correlated with Ph-VAS (ρ = 0.18, p = 0.003) and SLEDAI-2K (ρ = 0.14, p = 0.03), while anti-dsDNA was not correlated with SSC, SF-36, and LupusPRO, or Pt-VAS. In short-term and long-term, anti-dsDNA demonstrated no significant correlation with alterations in SSC, Pt-VAS, or Ph-VAS. Further, they did not show associations when SLE activities (SLEDAI-2K) were worsened.

**CONCLUSION:**

PROs at a time point and changes were difficult to be captured by anti-dsDNA. It is desirable to explore objective laboratory measures to evaluate PROs.

## INTRODUCTION

Systemic lupus erythematosus (SLE) is an autoimmune disease associated with diverse organs [1]. The accurate capture of disease activity is important for treating SLE [2]. Anti-dsDNA antibodies (Abs) titres have been reported to be correlated with SLE disease activity, especially during the induction of SLE remission [3, 4].

Currently, patient-reported outcomes (PROs), such as the SLE symptom checklist (SSC) [5], patient-visual analogue scale (Pt-VAS), LupusPRO [6], and Medical Outcomes Study Short Form-36 (SF-36) [7] are used in SLE practice, as well as objective measurements, such as the SLE Disease Activity Index 2000 (SLEDAI-2K) [8]. Since PROs are difficult to be captured by the other measurements, it is desirable to detect objective laboratory measures which are correlated with PROs and can capture the changes in PROs. Previously, no significant correlation has been reported between disease activity measures (SLEDAI-2K) and PRO (SF-36 health-related QoL) [9]. Regarding anti-dsDNA Ab, it has not been clearly understood whether PROs of SLE correlate well with anti-dsDNA Ab. Thus, there are questions to be investigated whether anti-dsDNA Ab captures PRO at a time point and changes in PRO.

Our research question is whether the anti-dsDNA Ab is correlated to the PRO (namely, Pt-VAS, SSC, LupusPRO, and SF-36) scores or not for SLE patients under treatment, especially under maintenance treatment. We analysed their associations under different conditions of timelines. First, we cross-sectionally examined the correlation between anti-dsDNA Ab and diverse measures of SLE, including the PROs (Pt-VAS, SSC, LupusPRO, and SF-36) and objective measures such as SLEDAI-2K and Physician (Ph)-VAS using a prospective cohort of SLE patients under treatment (study 1). Second, we examined the correlation between the changes of anti-dsDNA Ab and the changes of the PROs during both short- and long-term periods (study 2 and 3, respectively).

## METHODS

### Study population

**Figure 1:**
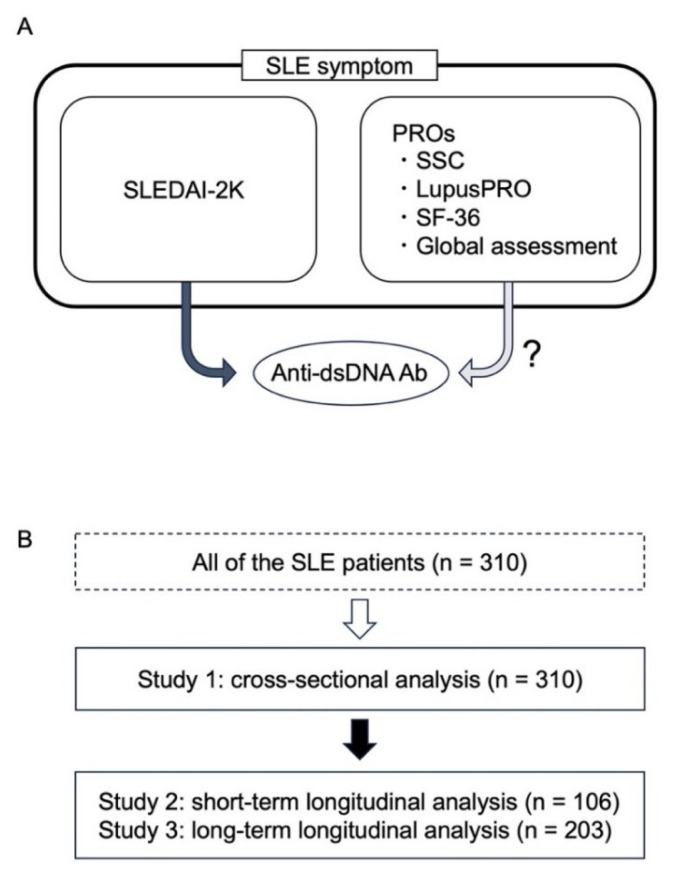
A, the research question; B, a flowchart of study design is shown. First, 310 patients with SLE were included, and the associations between their anti-dsDNA Ab titres with PROs, disease activities, and complements at one point visit were cross-sectionally analysed (study 1). Second, short-term longitudinal analyses of the associations between anti-dsDNA Ab with PROs and disease activity were conducted in 106 patients whose data were available (study 2). Third, long-term longitudinal analyses were performed with 203 patients whose data were available (study 3).

This study was conducted based on the Kyoto Lupus Cohort (KLC) between 1 April 2019 and 31 March 2020. The KLC is a single-centre cohort of patients with SLE who have visited Kyoto University Hospital since 2000 [10, 11]. All patients fulfilled the American College of Rheumatology (ACR) classification criteria in 1997 [12] or the Systemic Lupus International Collaborating Clinics (SLICC) classification criteria in 2012 [13]. After excluded patients under 18 years old, patients were included regardless of any treatments or disease activities. Patients available for the assessment items within certain periods were selected for both cross-sectional and longitudinal studies, as described below (Figure 1). The patients’ age, sex, comorbidities, and treatment were obtained from their electronic medical records in the hospital.

### Study design

#### Associations between anti-dsDNA antibodies and PROs in cross-sectional studies (study 1)

This cross-sectional study was conducted from September 2019 to June 2020 (n = 310). SLEDAI-2K, SSC, Ph-VAS, Pt-VAS, LupusPRO, and SF-36 version 2.0 were collected to evaluate the disease activity of SLE and general conditions (Figure 1). Correlations between anti-dsDNA Ab and PROs, as well as anti-dsDNA Ab and glucocorticoid dose (prednisolone equivalent) were evaluated. Correlations between comorbidities, such as Sjogren’s syndrome (SS) and antiphospholipid syndrome (APS), and PRO were also analysed. Further, correlations between anti-dsDNA Ab and disease activities and complement levels were evaluated. Additionally, correlation between each component of SLEDAI and PRO measures was evaluated. SLEDAI components were divided into 9 categories: seizure, psychosis, organic brain syndrome, visual disturbance, cranial nerve disorder, lupus headache, and cerebrovascular accidents as ‘neuropsychiatric’, vasculitis as ‘vasculitis’, arthritis and myositis as ‘musculoskeletal’, urinary casts, hematuria, proteinuria, and pyuria as ‘renal’, rash, alopecia, and mucosal ulcers as ‘mucocutaneous’, pleurisy and pericarditis as ‘serositis’, low complement and increased DNA binding as ‘serological’, fever as ‘fever’, and thrombocytopenia and leukopenia as ‘hematological’. Since serological category had been analysed, the other 8 categories were evaluated.

#### Associations between anti-dsDNA antibodies and PROs in longitudinal studies (study 2 and 3)

For the longitudinal study, data were acquired from patients with at least two data points for anti-dsDNA Ab, Pt-VAS, SSC score, SLEDAI-2K, and Ph-VAS available from July 2019 to July 2021 (n = 106) (Figure 1). The two points with the largest change in SLEDAI-2K were selected for patients with three or more data points. Since the changes of anti-dsDNA Ab and the changes of the disease activity (SLEDAI-2K) had relatively strong association, choosing the two time points in which SLEDAI-2K most increased would minimize the effect of anti-dsDNA titre fluctuation which is not clinically relevant.

Correlations among changes in anti-dsDNA Ab, Pt-VAS, and SSC over the short (3 months, study 2) and long (2 years, study 3) term were evaluated. To examine conditions of high activity of SLE and anti-DNA Abs, correlations were also evaluated under conditions of elevated SLEDAI-2K and elevated anti-dsDNA Ab levels.

#### Outcome measurements

We conducted a questionnaire-based survey (SLEDAI-2K, SSC, Ph-VAS, Pt-VAS, LupusPRO, and SF-36). The following scales were employed to evaluate the QOL: SF-36 [14], LupusPRO [6, 15], and SSC [5]. As for the SF-36, we used three summary components: physical (PCS), mental (MCS), and role/social component summary (RCS) [16]. We used norm-based scoring (NBS) points as the subscale scores, whose national standard score/standard deviation was adjusted to 50/10 based on the data. We excluded patients whose answer rates of any subscale domain were less than 50% from the analysis [17]. LupusPRO is an SLE-specific QOL assessment tool. It assesses the effects of SLE on the QOL by 12 subscale domains divided into two summary scores: health-related QOL (HRQOL) and non-health-related QOL (N-HRQOL). We excluded patients whose scores of any subscale domain were unable to be calculated. SSC is an SLE-specific QOL assessment tool that assesses the degree of 38 symptoms that are common in patients with SLE. It is specific to the patients’ subjective symptoms and assesses different aspects of the QOL from LupusPRO or the SF-36 [5, 11]. As for SSC, we used the Japanese version of the SSC (SSC-J) [11]. We complemented missing values by the mean of the other scores when the number of the missing items accounted for less than 20% [11]. We excluded patients whose answer rates were less than 80%. The higher scores of the SF-36 and LupusPRO indicate a better QOL, whereas higher scores of SSC indicate a poorer QOL.

#### Measurement of anti-dsDNA antibodies and complements

Anti-dsDNA Ab are measured by radioimmunoassay (LSI Medience) according to the manufacturer’s instructions (normal range, under 6 IU/mL). When the anti-dsDNA Ab was below the measurement limit, it was regarded as 0 IU/mL. Complement 3 (C3) and complement 4 (C4) were measured by turbidimetric immunoassay (TIA) according to the manufacturer’s instructions.

#### Ethical approval

This study was conducted in accordance with the Declaration of Helsinki and its amendments and was approved by the Kyoto University Hospital Ethics Committee (R1452). All the participants provided written informed consent.

#### Statistical analyses

Continuous variables were compared using Mann–Whitney U test, and categorical variables were compared using Fisher’s exact test. Spearman’s rank correlation analysis was conducted to determine correlations. To exclude the influence of anti-dsDNA Ab, two measurements derived from SLEDAI-2K were evaluated. One was modified SLEDAI-2K without the category of anti-dsDNA Ab. The other was clinical SLEDAI-2K, which omitted the category of anti-dsDNA Ab and complement [18]. P values < 0.05 were considered significant. JMP pro v16.1.0 was utilised for statistical analysis. Demographic data and clinical characteristics were described as the mean (standard deviation [SD]).

## RESULTS

### Study 1: associations between anti-dsDNA antibodies and PROs in cross-sectional studies

The total number of participants was 310. Almost all of the patients (90%) were treated with GC (Table I). The positivity of anti-dsDNA Ab was 34.2% at a time point, which was 17.6 ± 10.4 (mean ± SD) years after the onset of SLE (Table I). However, 84.8% of the patients was positive for anti-dsDNA Ab at diagnosis of SLE. Anti-Sm Ab and anti-RNP Ab were positive in 49.4% and 55.5% of patients, respectively. When the patients were classified as positive (n = 106) or negative (n = 204) for anti-dsDNA Ab, there were no significant differences in the SSC, Pt-VAS, mental component of SF-36, or role/social component of SF-36 between the two groups. Furthermore, the results of non-health related LupusPRO (43.9 ± 15.5 vs. 40.3 ± 13.8, p = 0.04), and physical component in SF-36 (46.0 ± 11.8 vs. 42.0 ± 13.0, p = 0.01) demonstrated slightly better outcomes in patients positive for anti-dsDNA Ab than in patients negative for anti-dsDNA Ab. On the other hand, levels of C3 (80.7 ± 20.2 mg/dL vs. 91.1 ± 21.1 mg/dL, p < 0.0001), and C4 (15.4 ± 6.97 mg/dL vs. 20.1 ± 7.81 mg/dL, p < 0.0001) were lower in patients positive for anti-dsDNA Ab compared with those negative for anti-dsDNA Ab (Table II). SLEDAI-2K (7.94 ± 5.20 vs. 4.56 ± 4.66, p < 0.0001), SLEDAI-2K without anti-dsDNA Ab (5.98 ± 5.21 vs. 4.56 ± 4.66, p = 0.016), and Ph-VAS (19.3 ± 16.0 vs. 14.3 ± 13.5, p = 0.005) were higher in patients positive for anti-dsDNA Ab (Table II).

The patients with hypocomplementemia had statistically significant difference, in comparison to those without hypocomplementemia, in their SF-36 role/social component summary (RCS) (51.6 ± 11.9 vs. 48.2 ± 13.0, p = 0.017) and SSC score (30.5 ± 19.2 vs. 36.9 ± 23.4, p = 0.040) (Supplemental Table I).

There were no significant differences in PROs (SF-36, LupusPRO, Pt-VAS, SSC) between patients with SS and patients without SS (Supplemental Table II). On the other hand, when we compared patients with APS and those without APS, there were differences between SF-36 mental component summary (MCS) and role/social component summary (43.91 ± 7.63 vs. 47.60 ± 9.18, p = 0.014 and 53.11 ± 12.49 vs. 48.72 ± 12.77, p = 0.038, respectively), which suggested that the patients with APS had worse MCS scores, while better RCS, than the patients without APS.

Next, we evaluated the correlation between the levels of anti-dsDNA Ab and SLE measurements at a time point. As a result, anti-dsDNA Ab slightly correlated with SLEDAI-2K (ρ = 0.29, p < 0.0001), Ph-VAS (ρ = 0.18, p = 0.003) (Table III). Clinical SLEDAI-2K was well correlated to original SLEDAI-2K and SLEDAI-2K without anti-dsDNA Ab (ρ = 0.94, p < 0.0001). However, no significant correlations were found between anti-dsDNA Ab and clinical SLEDAI-2K (ρ = 0.082, p = 0.17). On the other hand, no significant correlations were found between the SSC and anti-dsDNA Ab (ρ = −0.058, p = 0.34) or between the Pt-VAS and anti-dsDNA Ab (ρ = −0.07, p = 0.25). Furthermore, SF-36 (PCS and RCS) demonstrated slight improvements when anti-dsDNA Ab were elevated (ρ = 0.14, p = 0.022, and ρ = 0.12, p = 0.039, respectively).

Further, we evaluated the correlation between each SLEDAI component and PRO measures (Supplemental Table III). Pt-VAS was negatively correlated to SLEDAI hematological component (ρ = −0.13, p = 0.018). SSC had also negative correlation to the hematological component (ρ = −0.12, p = 0.034). These suggested that people with worse hematological component scores had favorable PRO scores. Contrary, LupusPRO (HRQOL) was significantly negatively correlated with SLEDAI musculoskeletal component (ρ = −0.11, p = 0.049) and SSC was significantly positively correlated with SLEDAI mucocutaneous component (ρ = 0.17, p = 0.0025). Both implied that worse disease activity resulted in worse PRO measures.

### Study 2: associations between anti-dsDNA antibodies with PROs and SLE disease activity in short observation periods

Alterations in anti-dsDNA Ab and PROs were evaluated in 106 patients for a period of 3 months. As a result, Δanti-dsDNA Ab demonstrated no significant correlation with ΔSSC (n = 106, ρ = −0.068, p = 0.49), ΔPt-VAS (ρ = −0.025, p = 0.80), and ΔPh-VAS (ρ = 0.11, p = 0.25) (Table IV). Furthermore, when they were additionally conditioned with elevated anti-dsDNA Ab and SLE activities (ΔSLEDAI-2K without anti-dsDNA Ab >0) (n = 39), Δanti-dsDNA Ab demonstrated no significant correlation with ΔSSC (ρ = −0.09, p = 0.58), ΔPt-VAS (ρ = −0.15, p = 0.34) and ΔPh-VAS (ρ = 0.07, p = 0.65). On the other hand, Δanti-dsDNA Ab significant correlated with ΔSLEDAI-2K (ρ = 0.37, p = 0.02), ΔSLEDAI-2K without anti-dsDNA Ab (ρ = 0.33, p = 0.04), and ΔcSLEDAI-2K (ρ = 0.38, p = 0.015).

### Study 3: associations between anti-dsDNA antibodies with PROs in long observation periods

We evaluated changes in anti-dsDNA Ab and PROs over a long observation period (825.0 ± 53.7 days, n = 203). As a result, Δanti-dsDNA Ab did not correlate with ΔPh-VAS (ρ = 0.14, p = 0.051), ΔPt-VAS (ρ = 0.028, p = 0.70), and ΔSSC (ρ = 0.081, p = 0.26), while Δanti-dsDNA Ab correlated with ΔSLEDAI-2K (ρ = 0.16, p = 0.027) (Table IV). Furthermore, when they were conditioned with elevated SLE activity and the elevation of anti-dsDNA Ab (n = 21), Δanti-dsDNA Ab did not demonstrate correlation with ΔPh-VAS, ΔPt-VAS, or ΔSSC.

## DISCUSSION

We examined the association between anti-dsDNA Ab and PROs in patients receiving maintenance therapy by both cross-sectional and longitudinal analyses. As a result, anti-dsDNA Ab did not correlate with PROs, while it correlated with SLEDAI-2K in a point and changes for a short-term period. It is well known that anti-dsDNA Ab is well correlated to SLE disease activity [19], especially in lupus nephritis [20]. And their association was supported by the cross reactivity to constituents of renal glomeruli in mice [21, 22]. However, the correlation of the PRO and the anti-dsDNA Ab was not as fully investigated as the basic research.

The study population was heterogeneous, as the patients were included in this study regardless of the treatments or disease activities. It was reflective of real world data, while the majority was with relatively low disease activity, as the mean SLEDAI2K was 5.72. Our result seemed not applicable to patients with acute-phase SLE, requiring intensive remission induction therapy.

As a cross-sectional analysis (study 1), patients positive for anti-dsDNA Ab demonstrated higher SLEDAI-2K and lower complement levels than patients negative for anti-dsDNA Ab, and anti-dsDNA Ab correlated with SLEDAI-2K and Ph-VAS scores at a time point. There were few reports that the correlation of anti-dsDNA Ab and Ph-VAS. However, it was acceptable that the patients positive for anti-dsDNA Ab had more severe SLE and physician marked higher Ph-VAS.

On the other hand, anti-dsDNA Ab were correlated with few PRO markers. Exceptionally, PCS in SF-36 and N-HRQOL in LupusPRO at a time point were better for patients positive for anti-dsDNA Ab than for those negative for anti-dsDNA Ab. The reports from BLYSS-52 and BLYSS-76, which were phase III trial of Belimumab, the anti-B lymphocyte stimulator (BLyS) antibody treatment, revealed that the treatment decreased anti-dsDNA Ab titre [23], and improved SF-36 PCS score [24]. Our result was inconsistent with the previous two research. It might be due to the difference in patient population homogeneity. As LupusPRO, there were fewer previous reports available.

It was reported that PROs were well correlated with SLE damage index (SDI), rather than SLEDAI [25]. Since our study population contained a high proportion of SLE patients with low disease activity, their PROs might reflect not the disease activity indices but the organ damage. It was regrettable that we did not have sufficient data to calculate SDI. Further investigation is warranted.

Anti-dsDNA Ab were inversely correlated with SF-36 (PCS and RCS). The results from longitudinal studies (study 2 and 3) also suggest that elevation of anti-dsDNA Ab can capture disease worsening under conditions with short observation periods (≤3 months), SLE activity with elevated anti-dsDNA Ab. On the other hand, it could not capture the changes of PROs. These results suggest that anti-dsDNA Ab reflects the patient’s SLE activity but cannot capture all SLE conditions.

To the best of our knowledge, no study has examined the correlation between anti-dsDNA Ab and PROs. Previously, no significant correlation has been reported between disease activity measures (SLEDAI-2K) and PRO (SF-36 health-related QoL) [25]. These findings suggest that PROs in patients under treatment would be difficult to be captured by anti-dsDNA Ab and SLEDAI-2K.

In this study, there were statistically significant differences in SF-36 MCS and RCS between the patients with APS and without it. It was reported that patients with APS had worse SF-36 MCS score [26]. However, RCS is relatively new concept and limited to Asian, so there were no reports that supported our finding. RCS could be affected by socioeconomical status. However, this was not included in the data available in our study.

Complement level was also an important serological marker in SLE. Our study revealed that only SF-36 RCS and SSC score were statistically significantly different between the patients with hypocomplementemia and without hypocomplementemia (RCS: 51.6 ± 11.9 vs. 48.2 ± 13.0, p = 0.017; SSC score: 30.5 ± 19.2 vs. 36.9 ± 23.4, p = 0.040). That was not consistent with the results of anti-dsDNA Ab. Since complement level was influenced by various factors other than SLE, such as hepatic disease, rheumatoid arthritis, or other collagen tissue disease, the differences in SF-36 RCS or SSC score between the patients with hypocomplementemia and the others might be derived from such diseases which affect complement level.

In this study, correlation between anti-dsDNA Ab and disease activity index were analysed in two-time course. In short time, there were statistically significant correlations between anti-dsDNA Ab and SLEDAI, or its modified version. However, in long time, such correlations were not detected. In long time analysis, there were only 20 patients in elevated SLEDAI/anti-dsDNA Ab group, which might affect the result. Other factors such as treatment change, multiorgan involvement, might also blunt the correlations of the anti-dsDNA Ab and SLEDAI.

Our study suggested that generally, the disease activity index, such as SLEDAI-2K, or anti-dsDNA Ab titre were not correlated to PRO. It is not clear whether clinicians should make decisions based on PRO. Baker et al. [27] reported that in RA, the Health Assessment Questionnaire (HAQ) score, pain visual analogue scale (VAS), and patient global assessment of disease activity (PtGl) VAS were statistically significantly correlated to MRI findings such as synovitis, osteitis, and bone erosion. The authors did not mention clinical decision-making. However, it is possible that patients with worse PRO need as intensive treatment as patients with worse disease activity do. Though that is not simply applicable to SLE treatment, there is possibility that the PRO reflects subtle disease activity that is not detectable in widely used disease activity index or laboratory tests.

Our result had some paradoxical points. In study 1, the patients with higher disease activity tended to report lower patient global VAS, though not statistically significant. Parodis et al. [28] reported that patient reported VAS of pain, fatigue, and overall SLE-related health state had all statistically significant positive correlation to clinical SLEDAI, unlike our result. We further analysed the correlation between PRO score and each SLEDAI category (Supplemental Table III). It revealed that Pt-VAS was negatively correlated to SLEDAI hematological component. Although hematological factors have relatively low weight (1 point) in SLEDAI, they might result in the paradoxical correlation between SLEDAI and Pt-VAS. Another possible explanation was SLE symptom subtype. Pisetsky et al. [29] claimed that SLE symptoms were divided into two types. Type 1 symptom included the classic signs and symptoms of inflammation such as nephritis, arthritis, vasculitis, and so on. Type 2 was non-inflammatory symptoms, (e.g., fatigue, widespread pain, and depression). Some of our study population might suffer from type 2 symptoms, whose SLEDAI would not be elevated but PRO markers would be affected.

There are several limitations in the current study. Anti-dsDNA Abs were negative in two thirds of all cases at study period, which might have influence on the results. However, 84.8% of the patients had positive anti-dsDNA Abs at least one time from their diagnoses to the study period. In fact, it was reported that the treatment of SLE decreased the anti-dsDNA Ab, as well as improved clinical signs and symptoms [23]. LupusPRO and SF-36 were not longitudinally evaluated. As SS and APS, there were limited number of patients whose PRO and anti-dsDNA Ab titre were available. Also, because of data unavailability, we could not investigate the detailed correlation of PRO measurements and the other objective parameter such as complement. In one-point analysis, there were some differences between patients with hypocomplementemia and patients without it. This is desired to be clarified with more comprehensive data collection. There was a patient bias due to a single-centre design. It might be a data bias that the missing values in PRO questionnaire were complemented by the mean of other values. To resolve these problems, more patients from multiple institutions with detailed data should be evaluated. Further, since this study was designed for patients with treatment, there might be a more dramatic change for patients with remission induction therapy, suggesting anti-DNA Abs may correlate with PROs in certain conditions.

In conclusion, it is difficult for anti-dsDNA Ab to reflect PROs in SLE, while the measurement of anti-dsDNA Ab at short intervals would be helpful in capturing SLE disease activity (SLEDAI-2K). PRO is understandable to show a different course from anti-dsDNA Ab and SLEDAI-2K in SLE practice. It is desirable to explore objective measures to evaluate PROs in SLE.

## Supplementary Data

**Supplemental Table I:** Patient reported outcome score comparison between SLE patients with or without hypocomplementemia

PRO outcome	Hypocomplementemia+ (N = 87)	Hypocomplementemia− (N = 223)	p value
SF-36			
PCS	45.62 ± 11.30	42.47 ± 13.17	0.090
MCS	48.03 ± 8.27	46.81 ± 9.43	0.55
RCS	51.57 ± 11.90	48.19 ± 12.98	0.016 *
LupusPRO			
HRQOL	71.53 ± 20.92	66.94 ± 20.92	0.073
N-HRQOL	41.65 ± 12.82	41.50 ± 15.14	0.98
SSC	30.48 ± 19.16	36.87 ± 23.43	0.040 *
Pt-VAS	34.48 ± 24.25	37.62 ± 24.28	0.33

Data are shown in mean ± SD.

* p < 0.05. Statistical analysis was performed by Mann–Whitney U test.

PRO, patient reported outcome; SF-36, 36-Item Short-Form Health Survey; PCS, physical component summary; MCS, mental component summary; RCS, role/social component summary; LupusPRO, lupus patient reported outcome tool; HRQOL, health-related quality of life; N-HRQOL, non-health-related quality of life; SLE, systemic lupus erythematosus; SSC, SLE symptom checklist; Pt-VAS, patient visual analogue scale.

**Supplemental Table II:** Patient reported outcome score comparison between SLE patients with or without Sjogren’s syndrome and anti-phospholipid syndrome

PRO outcome	SS+ (N = 36)	SS− (N = 270)	p value
SF-36			
PCS	39.72 ± 12.43	43.74 ± 12.76	0.069
MCS	47.83 ± 8.40	47.10 ± 9.21	0.80
RCS	50.52 ± 10.25	48.97 ± 13.05	0.80
LupusPRO			
HRQOL	68.49 ± 19.88	68.28 ± 21.19	0.97
N-HRQOL	44.88 ± 14.67	41.04 ± 14.48	0.084
SSC	39.54 ± 23.41	34.68 ± 22.42	0.22
Pt-VAS	43.11 ± 26.92	35.85 ± 23.93	0.13

**PRO outcome**	**APS+ (n = 30)**	**APS− (n = 277)**	**p value**

SF-36			
PCS	43.82 ± 8.67	43.38 ± 13.07	0.65
MCS	43.91 ± 7.63	47.60 ± 9.18	0.014 *
RCS	53.11 ± 12.49	48.72 ± 12.77	0.038 *
LupusPRO			
HRQOL	63.33 ± 20.86	69.02 ± 20.90	0.14
N-HRQOL	41.76 ± 17.18	41.39 ± 14.22	0.91
SSC	36.02 ± 21.21	34.88 ± 22.60	0.65
Pt-VAS	40.57 ± 24.77	36.23 ± 24.34	0.33

Data are shown in mean ± SD.

* p < 0.05. Statistical analysis was performed by Mann–Whitney U test.

PRO, patient reported outcome; SS, Sjogren’s syndrome; SF-36, 36-Item Short-Form Health Survey; PCS, physical component summary; MCS, mental component summary; RCS, role/social component summary; LupusPRO, lupus patient reported outcome tool; HRQOL, health-related quality of life; N-HRQOL, non-health-related quality of life; SLE, systemic lupus erythematosus; SSC, SLE symptom checklist; Pt-VAS, patient visual analogue scale.

**Supplemental Table III:** Patient reported outcome score comparison between each component of SLEDAI (serology was omitted)

PRO outcome	SLEDAI component	Spearman’s rho	p value
**3PCS**	vasculitis	−0.094	0.098
	fever	0.024	0.67
	renal	0.014	0.81
	neuropsychiatric	0.011	0.85
	mucocutaneous	−0.030	0.60
	serositis	−0.063	0.27
	hematological	0.038	0.51
	musculoskeletal	−0.096	0.091
**3MCS**	vasculitis	0.085	0.14
	fever	−0.056	0.32
	renal	−0.025	0.66
	neuropsychiatric	−0.024	0.67
	mucocutaneous	−0.0011	0.98
	serositis	−0.0003	0.99
	hematological	0.089	0.12
	musculoskeletal	−0.030	0.60
**3RCS**	vasculitis	0.080	0.16
	fever	−0.078	0.17
	renal	0.027	0.64
	neuropsychiatric	0.014	0.81
	mucocutaneous	0.023	0.69
	serositis	0.029	0.61
	hematological	0.081	0.16
	musculoskeletal	−0.0022	0.97
**LupusPRO (HRQOL)**	vasculitis	−0.022	0.70
	fever	−0.013	0.83
	renal	0.058	0.32
	neuropsychiatric	−0.026	0.66
	mucocutaneous	−0.098	0.092
	serositis	0.056	0.33
	hematological	0.046	0.43
	musculoskeletal	−0.11	0.049 *
**LupusPRO (N-HRQOL)**	vasculitis	−0.036	0.54
	fever	−0.053	0.37
	renal	−0.053	0.36
	neuropsychiatric	−0.11	0.056
	mucocutaneous	−0.0072	0.90
	serositis	−0.0007	0.99
	hematological	0.065	0.26
	musculoskeletal	−0.0088	0.88
**Pt-VAS**	vasculitis	0.0044	0.94
	fever	−0.012	0.84
	renal	0.013	0.82
	neuropsychiatric	0.06	0.30
	mucocutaneous	0.11	0.063
	serositis	−0.020	0.72
	hematological	−0.13	0.018 *
	musculoskeletal	0.048	0.40
**SSC**	vasculitis	0.078	0.17
	fever	−0.063	0.27
	renal	0.0075	0.90
	neuropsychiatric	0.089	0.12
	mucocutaneous	0.17	0.0025 *
	serositis	−0.057	0.33
	hematological	−0.12	0.034 *
	musculoskeletal	0.051	0.38

* p < 0.05.

PRO, patient reported outcome; PCS, physical component summary; MCS, mental component summary; RCS, role/social component summary; LupusPRO, lupus patient reported outcome tool; HRQOL, health-related quality of life; N-HRQOL, non-health-related quality of life; Pt-VAS, patient visual analogue scale; SLE, systemic lupus erythematosus; SSC, SLE symptom checklist.

## Figures and Tables

**Table I:** Clinical characteristics of patients at a time point in the maintenance therapy (n = 310)

Variable	Results
**At a time point**	
Sex (female)	284 (91.6%)
Age (y)	48.9 ± 13.8
Disease duration (y)	17.6 ± 10.4
Positive for anti-dsDNA antibody (RIA)	106 (34.2%)
Anti-dsDNA antibody (RIA) (U/mL)	8.29 ± 15.2
SSC	35.1 ± 22.5
Patient-VAS	36.7 ± 24.3
Physician-VAS	16.0 ± 14.6
SF-36 (PCS)	43.4 ± 12.7
SF-36 (MCS)	47.1 ± 9.12
SF-36 (RCS)	49.1 ± 12.8
LupusPRO (HRQOL)	68.2 ± 21.0
LupusPRO (N-HRQOL)	41.5 ± 14.5
C3 (mg/dL)	80.7 ± 20.2
C4 (mg/dL)	15.4 ± 6.97
SLEDAI-2K	5.72 ± 5.10
SLEDAI-2K w/o anti-DNA	5.05 ± 4.89
cSLEDAI-2K	4.47 ± 4.87
GC dose (prednisolone equivalent, mg/day)	7.24 ± 11.8
GC use	280 (90.0%)
Hydroxychloroquine	78 (25.2%)
Azathioprine	38 (12.3%)
Belimumab	24 (7.7%)
Cyclosporin A	17 (5.5%)
Tacrolimus	94 (30.3%)
Mycophenolate mofetil	40 (12.9%)
Mizoribine	17 (5.5%)
Methotrexate	13 (4.2%)

**At the diagnosis**	
Positive for anti-dsDNA	263 (84.8%)
Positive for anti-Sm	153 (49.4%)
Positive for anti-RNP	172 (55.5%)

Nominal variables are shown as n (%). Continuous variables are shown as mean ± standard deviation.

y, years; SLE, systemic lupus erythematosus; SSC, SLE symptom checklist; VAS, visual analogue scale; SF-36, 36-Item Short Form Survey; PCS, physical component summary; MCS, mental component summary; RCS, role/social component summary; LupusPRO, lupus patient reported outcome tool; HRQOL, health-related quality of life; N-HRQOL, non-health-related quality of life; C, complement; SLEDAI-2K, systemic lupus erythematosus disease activity index-2000; cSLEDAI-2K, clinical systemic lupus erythematosus disease activity index-2000; GC, glucocorticoid; RNP, ribonucleoprotein.

**Table II:** Comparison of measurements between patients positive and negative for anti-dsDNA antibodies at a time point (n = 310)

	Positive for anti-dsDNA Ab (n = 106)	Negative for anti-dsDNA Ab (n = 204)	P value
Sex (female)	98 (92.5%)	186 (91.2%)	0.83
Age (y)	47.4 ± 13.8	49.8 ± 13.7	0.15
Disease duration (y)	16.5 ± 9.72	18.2 ± 10.7	0.20
GC use	95 (89.6%)	185 (90.7%)	0.84
GC dose (mg/day)	7.33 ± 10.5	7.23 ± 12.4	0.95
SSC	33.3 ± 21.2	36.1 ± 23.1	0.29
Patient-VAS	34.6 ± 22.2	37.8 ± 25.3	0.27
Physician-VAS	19.3 ± 16.0	14.3 ± 13.5	0.005*
SF-36 (PCS)	46.0 ± 11.8	42.0 ± 13.0	0.01*
SF-36 (MCS)	47.6 ± 0.89	46.9 ± 0.64	0.55
SF-36 (RCS)	50.2 ± 11.8	48.6 ± 13.2	0.28
LupusPRO (HRQOL)	68.4 ± 21.5	68.2 ± 20.8	0.95
LupusPRO (N-HRQOL)	43.9 ± 15.5	40.3 ± 13.8	0.04*
C3 (mg/dL)	80.7 ± 20.2	91.1 ± 21.1	<0.0001*
C4 (mg/dL)	15.4 ± 6.97	20.1 ± 7.81	<0.0001*
SLEDAI-2K	7.94 ± 5.20	4.56 ± 4.66	<0.0001*
SLEDAI-2K w/o anti-DNA	5.98 ± 5.21	4.56 ± 4.66	0.016*
cSLEDAI-2K	5.16 ± 5.21	4.11 ± 4.65	0.068

Nominal variables are shown as n (%). Continuous variables are shown as mean ± standard deviation. Statistical analysis was conducted by Fisher’s exact test for nominal valuables and Mann–Whitney U test for continuous variables.

* p < 0.05.

y, years; GC, glucocorticoid; SLE, systemic lupus erythematosus; SSC, SLE symptom checklist; VAS, visual analogue scale; SF-36, 36-Item Short Form Survey; PCS, physical component summary; MCS, mental component summary; RCS, role/social component summary; LupusPRO, lupus patient reported outcome tool; HRQOL, health-related quality of life; N-HRQOL, non-health-related quality of life; C, complement; SLEDAI-2K, systemic lupus erythematosus disease activity index-2000; cSLEDAI-2K, clinical systemic lupus erythematosus disease activity index-2000.

**Table III:** Correlations between anti-dsDNA antibodies with PROs in a time point

	ρ	P value
SSC	−0.058	0.34
Ph-VAS	0.18	0.003*
Pt-VAS	−0.07	0.25
SF-36 (PCS)	0.14	0.022*
SF-36 (MCS)	−0.018	0.77
SF-36 (RCS)	0.12	0.039*
LupusPRO (HRQOL)	0.023	0.71
LupusPRO (N-HRQOL)	−0.080	0.19
C3	−0.24	<0.0001*
C4	−0.32	<0.0001*
SLEDAI-2K	0.29	<0.0001*
SLEDAI-2K w/o anti-dsDNA Ab	0.13	0.03*
cSLEDAI-2K	0.082	0.17

ρ: Spearman’s rank correlation coefficient.

* p < 0.05.

SLE, systemic lupus erythematosus; SSC, SLE symptom checklist; Ph-VAS, physician visual analogue scale; Pt-VAS, patient visual analogue scale; SF-36, 36-Item Short-Form Health Survey; PCS, physical component summary; MCS, mental component summary; RCS, role/social component summary; LupusPRO, lupus patient reported outcome tool; HRQOL, health-related quality of life; N-HRQOL, non-health-related quality of life; C, complement; SLEDAI-2K, systemic lupus erythematosus disease activity index-2000; cSLEDAI-2K, clinical systemic lupus erythematosus disease activity index-2000.

**Table IV:** Correlations between alterations in anti-dsDNA antibodies and measurements in short-/long-term observations

	All		Elevated SLEDAI-2K and anti-dsDNA Ab	

	ρ	p	ρ	p
**Short-term** (study 2)	(n = 106)		(n = 39)	
ΔPt-VAS	−0.025	0.80	−0.15	0.34
ΔPh-VAS	0.11	0.25	0.07	0.65
ΔSSC	−0.068	0.49	−0.09	0.58
ΔSLEDAI-2K	0.10	0.31	0.37	0.02*
ΔSLEDAI-2K w/o anti-dsDNA Ab	0.07	0.47	0.33	0.04*
ΔcSLEDAI-2K	0.080	0.42	0.38	0.015*
ΔGC	−0.0066	0.95	0.082	0.62

**Long-term** (study 3)	(n = 203)		(n = 21)	
ΔPt-VAS	0.028	0.70	−0.17	0.49
ΔPh-VAS	0.14	0.051	−0.082	0.72
ΔSSC	0.081	0.26	−0.053	0.82
ΔSLEDAI-2K	0.16	0.027*	0.19	0.40
ΔSLEDAI-2K w/o anti-dsDNA Ab	0.087	0.21	−0.036	0.88
ΔcSLEDAI-2K	0.066	0.35	−0.051	0.82
ΔGC	−0.0010	0.99	−0.16	0.49

Results of short-term observations (study 2) are shown for total population (n = 106), and elevated SLEDAI-2K (without antidsDNA Ab) and elevated anti-DNA (n = 40). Results of long-term observations (study 3) are shown for total population (n = 203), and patients with elevated SLEDAI-2K (without anti-dsDNA Ab) and elevated anti-DNA (n = 21).

ρ: Spearman’s rank correlation coefficient.

* p < 0.05.

Pt-VAS, patient visual analogue scale; Ph-VAS, physician visual analogue scale; SLE, systemic lupus erythematosus; SSC, SLE symptom checklist; SLEDAI-2K, systemic lupus erythematosus disease activity index-2000; cSLEDAI-2K, clinical systemic lupus erythematosus disease activity index-2000; GC, glucocorticoid.

## Data Availability

The data supporting the findings of this study are available from the corresponding author upon reasonable request.

## References

[1] Anders HJ, Saxena R, Zhao MH, Parodis I, Salmon JE, Mohan C (2020). Lupus nephritis. Nat Rev Dis Primers.

[2] Stoll T, Sutcliffe N, Mach J, Klaghofer R, Isenberg DA (2004). Analysis of the relationship between disease activity and damage in patients with systemic lupus erythematosus—a 5-yr prospective study. Rheumatology (Oxford).

[3] Pisetsky DS (2016). Anti-DNA antibodies--quintessential biomarkers of SLE. Nat Rev Rheumatol.

[4] Schur PH, Sandson J (1968). Immunologic factors and clinical activity in systemic lupus erythematosus. N Engl J Med.

[5] Grootscholten C, Ligtenberg G, Derksen RHWM, Schreurs KMG, de Glas-Vos JW, Hagen EC (2003). Health-related quality of life in patients with systemic lupus erythematosus: Development and validation of a lupus specific symptom checklist. Qual Life Res.

[6] Jolly M, Pickard AS, Block JA, Kumar RB, Mikolaitis RA, Wilke CT (2012). Disease-Specific Patient Reported Outcome Tools for Systemic Lupus Erythematosus. Semin Arthritis Rheum.

[7] Ware JEJ (2000). SF-36 Health Survey Update. Spine.

[8] Gladman DD, Ibañez D, Urowitz MB (2002). Systemic lupus erythematosus disease activity index 2000. J Rheumatol.

[9] Ghirardello A, Villalta D, Morozzi G, Afeltra A, Galeazzi M, Gerli R (2011). Diagnostic accuracy of currently available anti-double-stranded DNA antibody assays. An Italian multicentre study. Clin Exp Rheumatol.

[10] Takase Y, Iwasaki T, Doi H, Tsuji H, Hashimoto M, Ueno K (2021). Correlation between irreversible organ damage and the quality of life of patients with systemic lupus erythematosus: The Kyoto Lupus Cohort survey. Lupus.

[11] Doi H, Ohmura K, Tabuchi Y, Hashimoto M, Takase Y, Inaba R (2021). Validation and verification of the Japanese version of the systemic lupus erythematosus symptom checklist for patient quality of life. Lupus.

[12] Hochberg MC (1997). Updating the American College of Rheumatology revised criteria for the classification of systemic lupus erythematosus. Arthritis Rheum.

[13] Petri M, Orbai AM, Alarcón GS, Gordon C, Merrill JT, Fortin PR (2012). Derivation and validation of the Systemic Lupus International Collaborating Clinics classification criteria for systemic lupus erythematosus. Arthritis Rheum.

[14] Fukuhara S, Bito S, Green J, Hsiao A, Kurokawa K (1998). Translation, adaptation, and validation of the SF-36 Health Survey for use in Japan. J Clin Epidemiol.

[15] Inoue M, Shiozawa K, Yoshihara R, Yamane T, Shima Y, Hirano T (2017). The Japanese LupusPRO: A cross-cultural validation of an outcome measure for lupus. Lupus.

[16] Suzukamo Y, Fukuhara S, Green J, Kosinski M, Gandek B, Ware JE (2011). Validation testing of a three-component model of Short Form-36 scores. J Clin Epidemiol.

[17] Fukuhara S (2011). Manual of SF-36v2 Japanese version. (No Title).

[18] Parodis I, Emamikia S, Gomez A, Gunnarsson I, van Vollenhoven RF, Chatzidionysiou K (2019). Clinical SLEDAI-2K zero may be a pragmatic outcome measure in SLE studies. Expert Opin Biol Ther.

[19] ter Borg EJ, Horst G, Hummel EJ, Limburg PC, Kallenberg CG (1990). Measurement of increases in anti-double-stranded DNA antibody levels as a predictor of disease exacerbation in systemic lupus erythematosus. A long-term, prospective study. Arthritis Rheum.

[20] Pan N, Amigues I, Lyman S, Duculan R, Aziz F, Crow MK (2014). A surge in anti-dsDNA titer predicts a severe lupus flare within six months. Lupus.

[21] Deocharan B, Qing X, Lichauco J, Putterman C (2002). Alpha-actinin is a cross-reactive renal target for pathogenic anti-DNA antibodies. J Immunol.

[22] Amital H, Heilweil M, Ulmansky R, Szafer F, Bar-Tana R, Morel L (2005). Treatment with a laminin-derived peptide suppresses lupus nephritis. J Immunol.

[23] Furie R, Rovin BH, Houssiau F, Malvar A, Teng YKO, Contreras G (2020). Two-Year, Randomized, Controlled Trial of Belimumab in Lupus Nephritis. N Engl J Med.

[24] Strand V, Levy RA, Cervera R, Petri MA, Birch H, Freimuth WW (2014). Improvements in health-related quality of life with belimumab, a B-lymphocyte stimulator-specific inhibitor, in patients with autoantibody-positive systemic lupus erythematosus from the randomised controlled BLISS trials. Ann Rheum Dis.

[25] Baba S, Katsumata Y, Okamoto Y, Kawaguchi Y, Hanaoka M, Kawasumi H (2018). Reliability of the SF-36 in Japanese patients with systemic lupus erythematosus and its associations with disease activity and damage: a two-consecutive year prospective study. Lupus.

[26] Zuily S, Rat AC, Regnault V, Kaminsky P, Mismetti P, Ninet J (2015). Impairment of quality of life in patients with antiphospholipid syndrome. Lupus.

[27] Baker JF, Conaghan PG, Emery P, Baker DG, Ostergaard M (2017). Relationship of patient-reported outcomes with MRI measures in rheumatoid arthritis. Ann Rheum Dis.

[28] Parodis I, Stephens T, Dominicus A, Eek D, Sjöwall C (2024). Lupus Low Disease Activity State and organ damage in relation to quality of life in systemic lupus erythematosus: a cohort study with up to 11 years of follow-up. Rheumatology (Oxford).

[29] Pisetsky DS, Clowse MEB, Criscione-Schreiber LG, Rogers JL (2019). A Novel System to Categorize the Symptoms of Systemic Lupus Erythematosus. Arthritis Care Res (Hoboken).

